# A tool for discovering drug sensitivity and gene expression associations in cancer cells

**DOI:** 10.1371/journal.pone.0176763

**Published:** 2017-04-28

**Authors:** Yong Qin, Anthony P. Conley, Elizabeth A. Grimm, Jason Roszik

**Affiliations:** 1 Department of Melanoma Medical Oncology, The University of Texas MD Anderson Cancer Center, Houston, Texas, United States of America; 2 Department of Sarcoma Medical Oncology, The University of Texas MD Anderson Cancer Center, Houston, Texas, United States of America; 3 Department of Genomic Medicine, The University of Texas MD Anderson Cancer Center, Houston, Texas, United States of America; Wayne State University, UNITED STATES

## Abstract

The sensitivity of cancer cells to anticancer drugs is a crucial factor for developing effective treatments. However, it is still challenging to precisely predict the effectiveness of therapeutics in humans within a complex genomic and molecular context. We developed an interface which allows the user to rapidly explore drug sensitivity and gene expression associations. Predictions for how expression of various genes affect anticancer drug activity are available for all genes for a set of therapeutics based on data from various cell lines of different origin in the Cancer Cell Line Encyclopedia and the Genomics of Drug Sensitivity in Cancer projects. Our application makes discovery or validation of drug sensitivity and gene expression associations efficient. Effectiveness of this tool is demonstrated by multiple known and novel examples.

## Introduction

*In silico* prediction of drug efficacy and resistance significantly increases efficiency of drug discovery. Multiple computational tools have been developed to help identify potential indications for drugs using molecular features that are now available in large public databases [1]. Drug sensitivity information on cell lines is one of these features which is frequently used to identify potential therapeutics for cancer and other diseases. However, drug effectiveness is not only determined by the expression of the direct target, but it also depends on genomic background and other molecules that affect the biological context of the drug-target interaction [2]. To improve drug effectiveness and identify potential combination therapeutics for cancer, it is essential to examine the effects of different genes on drug activity within a large biological context. This can help to identify key factors correlating with drug response that may be secondary targets. A way to achieve this goal is identifying genes that show an association with response/resistance to drugs by screening of cell lines.

The databases of Cancer Cell Line Encyclopedia (CCLE) [3] and Genomics of Drug Sensitivity in Cancer (GDSC) [4] projects contain gene expression levels from next generation sequencing and also drug screening data for a large number of cancer cell lines. In order to efficiently examine relationships of a given gene and the sensitivity of cancer cells to specific anticancer drugs, we created an interface that can be rapidly queried to identify potentially relevant genes/targets associated with drug effectiveness in specific cancer types based on the genomic and pharmacologic data of cancer cell lines in the CCLE and GDSC. Moreover, the results from one data set can be used as validation data for the other.

Using this method, we recently found that higher expression of HGF, MET, and VEGF-A genes correlates with lower sensitivity to a BRAF(V600E) inhibitor in melanoma cells [5]. The positive correlation of HGF, MET, and VEGF-A expression and PLX4720 EC50 indicated that hypoxia-driven upregulation of these genes results in increased resistance to PLX4720 in melanoma. Our *in vitro* drug studies confirmed that high level of HGF/MET signaling correlates with low sensitivity to a BRAF(V600E) inhibitor in melanoma [5].

Our application makes it easy to identify such associations, and it provides detailed correlation analyses for hypothesis generation or validation purposes. The tool is freely available as a web interface, and it can also be downloaded and used in the Tableau Desktop software.

## Results and discussion

In our previous study [5], we have successfully used this approach to identify HGF, MET, and VEGF-A expression correlations with resistance to a BRAF(V600E) inhibitor, which was experimentally confirmed and has clinically relevant implications. We also used this method to analyze the role of iNOS in pancreatic cancer [6]. Here we present two analyses showing novel relationships between two relevant genes and multiple anticancer drugs in various cancers. The tool is freely available at: https://public.tableau.com/profile/jason.roszik#!/vizhome/CCLE_GDSC_correlations/CCLE_GDSC

### NQO1 expression correlates with 17-AGG activity

**Fig 1:**
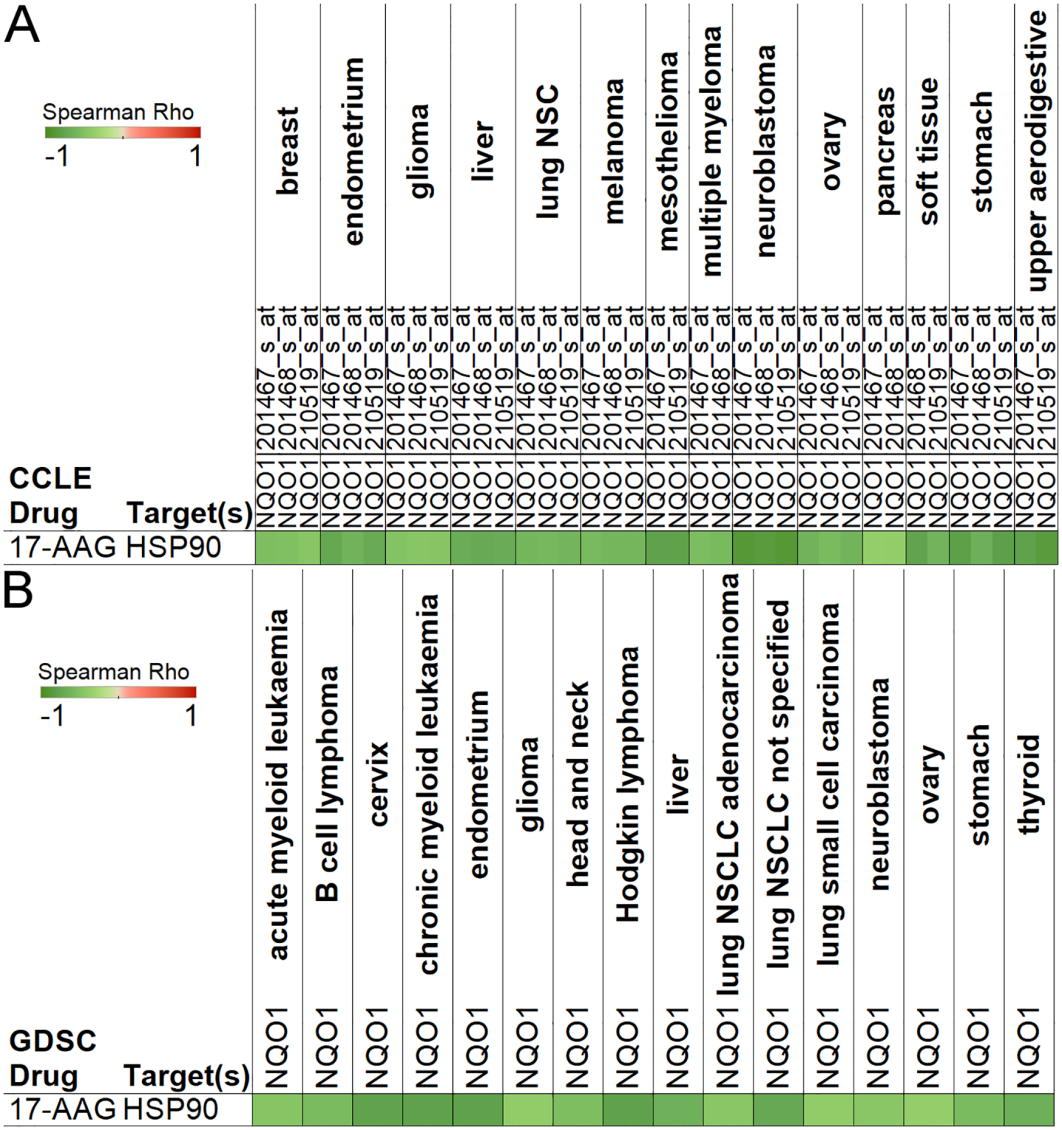
NQO1 expression correlates with 17-AAG effectiveness in multiple cancer types. Green color represents negative Spearman’s rank correlation coefficients between NQO1expression and 17-AAG EC50 in the CCLE (A) and IC50 (EC50 was not available) in GDSC (B). These results indicate that 17-AAG works better when NQO1 expression is higher. Only p < 0.05 correlations are shown.

Heat shock protein 90 (Hsp90) is a molecular chaperone which has been successfully targeted in pre-clinical and clinical models to inhibit tumor growth. However, the optimal use of Hsp90 inhibitors is still to be determined in cancer patients [7]. It was recently shown that NAD(P)H:quinone dehydrogenase 1 (NQO1) expression and the Hsp90 inhibitor 17-AAG sensitivity are inversely correlated in melanoma cells [8]. Furthermore, 17-AAG sensitivity was found to be related to NQO1 protein levels and enzymatic activity in pancreatic and colorectal cancer cells [9]. These studies indicate that high levels NQO1 expression may sensitize cancer cells to 17-AAG. As shown in Fig 1, the analysis using our tool confirms that NQO1 expression negatively correlates with the EC50 of 17-AAG in melanoma and in pancreatic cancer cells in the CCLE. Moreover, this association was also found in various other cancer cell lines such as breast, liver, brain, ovary, and lung cancers, which have not been reported. Consistently, this negative correlation was confirmed in seven cancer types, endometrium, glioma, liver, non-small cell lung cancer, neuroblastoma, ovary, and stomach, in both CCLE and GDSC data sets (Fig 1). Our results point to the application of NQO1 as a predictive marker in a wide range of cancers to estimate effectiveness of Hsp90 inhibitors.

### Effect of VEGFA expression on multiple anticancer drugs’ sensitivity

**Fig 2:**
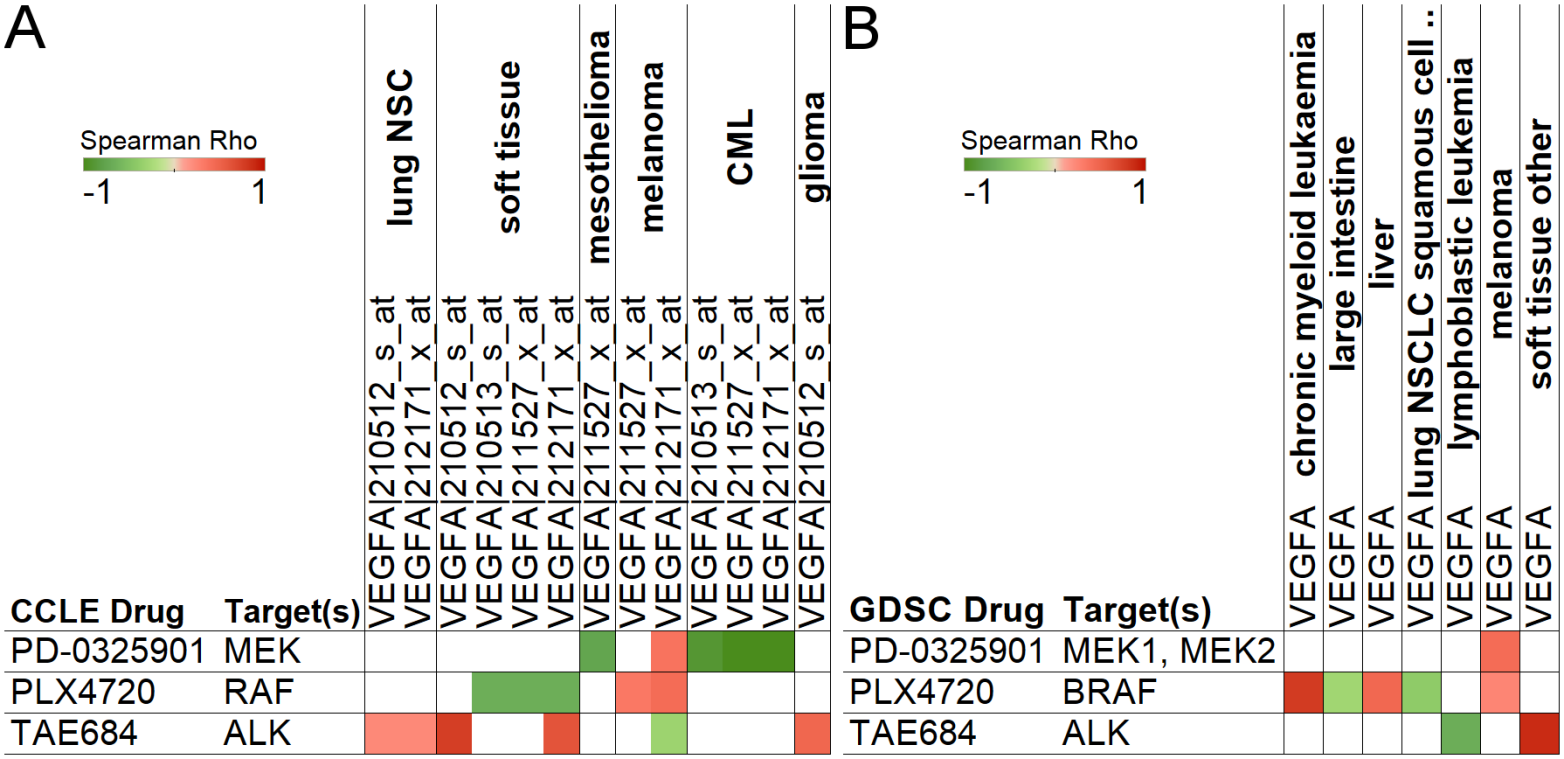
VEGFA expression correlates with drug sensitivity. Green and red colors represent negative and positive Spearman’s rank correlation coefficients between VEGFA levels and different anticancer drugs’ IC50 in the CCLE (A) and GDSC (B). A positive correlation with IC50 means that the drug is less effective; a negative correlation indicates that it is more effective when VEGFA expression is higher. p < 0.05 correlations are displayed.

Vascular endothelial growth factor A (VEGF-A) is a crucial signal protein which stimulates vasculogensis and angiogenesis. The overexpression of VEGFA has been associated with tumor progression and poor outcome in cancer patients [10]. VEGF-A production can be induced in cells under hypoxia, and hypoxia-inducible factor-1(HIF-1) can stimulate the transcription of VEGFA [10]. Hypoxia plays an important role in tumor progression and development of therapy resistance. As a result of hypoxia driven context changes, cancer cells under hypoxia change their sensitivity to drugs. Therapeutics have been developed to target VEGF-A, such as bevacizumab, which was approved by the FDA in 2004. However, only about 10–15% of patients benefit from bevacizumab therapy, and biomarkers for bevacizumab efficacy are not yet known [10]. Thus, it is important to examine the effect of VEGFA expression on other cancer therapeutics, as this may provide suggestions for combination therapies with VDGF-A inhibitors. As shown in Fig 2A of the CCLE data set, VEGFA expression positively correlates with IC50 of TAE684 in non-small cell lung cancer (NSCLC) and soft tissue tumor cell lines, indicating that high levels of VEGFA expression correlates with resistance to the TAE684 ALK inhibitor. Furthermore, this positive correlation was also confirmed in soft tissue cancer cells in the GDSC data set (Fig 2B). Intriguingly, a study by Kogita and colleagues showed that hypoxia induced resistance to ALK inhibitors [11], which provides additional support to suggest that hypoxia-driven upregulation of VEGF-A may contribute to ALK inhibitor resistance. In both CCLE and GDSC data sets, VEGFA expression positively correlates with IC50 of PD-0325901 (MEK inhibitor) and PLX4720 (BRAF(V600E) inhibitor) in melanoma cells (Fig 2A and 2B). Since MEK is the direct downstream mediator of BRAF signaling, our discovery further implicates that the overexpression of VEGF-A may be a crucial hypoxic factor contributing to the enhanced resistance to blockade of aberrant BRAF(V600E) signaling in melanoma. Interestingly, VEGFA expression can be either a potential positive or negative factor in different cancer types in responding to different treatments. For example, there are negative correlations between VEGFA expression and PLX4720 in soft tissue, large intestine, and non-small cell lung cancer cells (Fig 2A and 2B). These results indicate that VEGFA may play different roles in different cancer cells when using VEGF-A inhibitors, and it need to be carefully examined when combining with ALK, RAF, or MEK inhibitors in various cancers.

## Methods

IC50 and EC50 values, that are commonly used to evaluate drug effectiveness, were available in the Cancer Cell Line Encyclopedia (CCLE) project [3] for 24 well-known targeted therapeutics. Furthermore, IC50 results for 265 drugs can be downloaded from the Genomics of Drug Sensitivity in Cancer (GDSC) database [4]. More than one thousand cancer cell lines derived from various tissues that cover most common cancer types were tested with these therapeutics. Cell line information, gene expression, and drug screening data were downloaded from the website of the CCLE and GDSC projects.

Spearman’s rank correlation coefficients were calculated using the R software. To use only the robust correlations, we included only the correlations with p < 0.05. The visualization interface was created using the Tableau software [12]. In the web interface, correlation coefficients and p-values are shown when moving the mouse over areas of interest.

## Conclusions

We have developed an interface to identify potentially relevant targets associated with drug effectiveness in multiple cancer types based on genomic and pharmacologic data of cancer cell lines. By facilitating the identification of potential markers and targets for combination therapies this tool is intended to make drug development more cost-efficient and increase the likelihood for finding new targets.
